# Accuracy of Machine Learning Assisted Detection of Keratoconus: A Systematic Review and Meta-Analysis

**DOI:** 10.3390/jcm11030478

**Published:** 2022-01-18

**Authors:** Ke Cao, Karin Verspoor, Srujana Sahebjada, Paul N. Baird

**Affiliations:** 1 Centre for Eye Research Australia, Melbourne, VIC 3002, Australia; kcao1@student.unimelb.edu.au (K.C.); srujana.sahebjada@unimelb.edu.au (S.S.); 2 Department of Surgery, Ophthalmology, The University of Melbourne, Melbourne, VIC 3002, Australia; 3 School of Computing Technologies, RMIT University, Melbourne, VIC 3000, Australia; karin.verspoor@rmit.edu.au; 4 School of Computing and Information Systems, The University of Melbourne, Melbourne, VIC 3010, Australia

**Keywords:** keratoconus, diagnosis, early detection, artificial intelligence, machine learning, reporting completeness

## Abstract

(1) Background: The objective of this review was to synthesize available data on the use of machine learning to evaluate its accuracy (as determined by pooled sensitivity and specificity) in detecting keratoconus (KC), and measure reporting completeness of machine learning models in KC based on TRIPOD (the transparent reporting of multivariable prediction models for individual prognosis or diagnosis) statement. (2) Methods: Two independent reviewers searched the electronic databases for all potential articles on machine learning and KC published prior to 2021. The TRIPOD 29-item checklist was used to evaluate the adherence to reporting guidelines of the studies, and the adherence rate to each item was computed. We conducted a meta-analysis to determine the pooled sensitivity and specificity of machine learning models for detecting KC. (3) Results: Thirty-five studies were included in this review. Thirty studies evaluated machine learning models for detecting KC eyes from controls and 14 studies evaluated machine learning models for detecting early KC eyes from controls. The pooled sensitivity for detecting KC was 0.970 (95% CI 0.949–0.982), with a pooled specificity of 0.985 (95% CI 0.971–0.993), whereas the pooled sensitivity of detecting early KC was 0.882 (95% CI 0.822–0.923), with a pooled specificity of 0.947 (95% CI 0.914–0.967). Between 3% and 48% of TRIPOD items were adhered to in studies, and the average (median) adherence rate for a single TRIPOD item was 23% across all studies. (4) Conclusions: Application of machine learning model has the potential to make the diagnosis and monitoring of KC more efficient, resulting in reduced vision loss to the patients. This review provides current information on the machine learning models that have been developed for detecting KC and early KC. Presently, the machine learning models performed poorly in identifying early KC from control eyes and many of these research studies did not follow established reporting standards, thus resulting in the failure of these clinical translation of these machine learning models. We present possible approaches for future studies for improvement in studies related to both KC and early KC models to more efficiently and widely utilize machine learning models for diagnostic process.

## 1. Introduction

Corneal diseases are the second largest cause of blindness worldwide, behind only cataract in overall importance [1]. Keratoconus (KC), one of the most common corneal conditions, is characterized by bilateral, progressive corneal thinning that results in an abnormally steep cornea, and decreased vision [2]. The disease primarily affects young adults and children [3]. Globally KC patients comprise the second largest group of patients requiring corneal transplants, and there are thus associated risks of surgical complications and immune rejection of the graft [4,5].

In the medical field, the recent availability of biomedical data has led to the advent of the big data era [6,7], creating opportunities for more comprehensive data-informed decision making. The challenge for the clinician has expanded beyond data collection to encompass the interpretation of a greater amount of information. Artificial intelligence (AI) is the ability of a machine to learn and display intelligence [8]. Machine learning methods represent a branch of AI where computational algorithms can be used to process and identify patterns in large amounts of data at a scale that is beyond the ability of humans to synthesize. Through advanced pattern mining, innovative detection solutions, referred to as automatic detection models, can be based on these massive amounts of data [9].

Increased detailed data about the cornea resulting from corneal topography and tomography systems are essential for diagnosing KC. They are especially useful for detecting early indications of KC, prior to the development of typical KC clinical characteristics [10]. Corneal tomography provides parameters and images [11], which are laborious to analyze manually. This has also been a motivating force in the use of machine learning for KC detection since 1995 [12], with a growing volume of machine learning research being conducted in KC detection over the following years.

There is no existing study that summarizes the use of machine learning in KC, identifies limitations, and makes recommendations for future directions. The aim of this study and meta-analysis was to systematically review all currently available literature to determine accuracy (through the use of pooled sensitivity and specificity) of machine learning in the detection of KC, addressing this knowledge gap.

## 2. Materials and Methods

### 2.1. Literature Search Strategy

**Figure 1:**
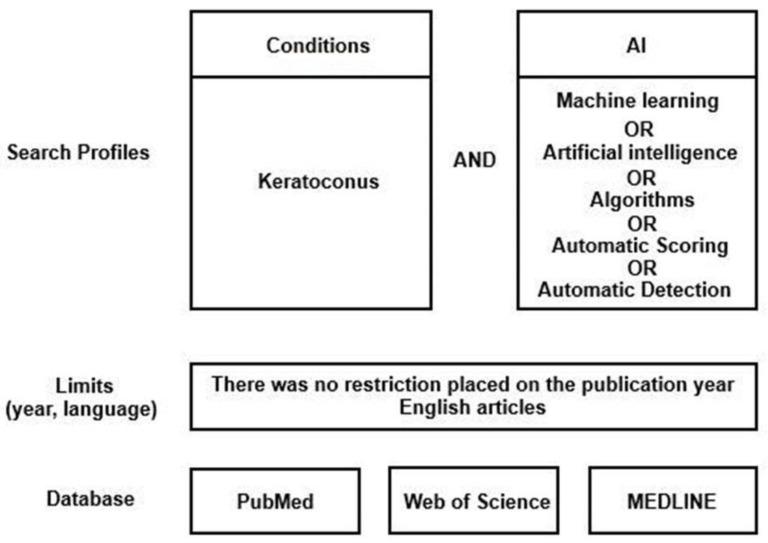
The search strategy used in the present study.

A web-based systematic literature search was performed for articles published from inception through 28 February 2021, on PubMed, Web of Science, and MEDLINE (Figure 1). Database searches were supplemented by hand-search and grey literature search techniques to ensure all publications in this field were included. The protocol for this systematic review was registered on PROSPERO (registration number CRD42021237167).

PubMed, Web of Science, and MEDLINE were interrogated using search strings pertaining to keratoconus and machine learning. Key words were used by search engine and designated filters according to PRISMA (Preferred Reporting Items for Systematic Reviews and Meta-Analyses) guidelines (Figure 1). The search string was ((keratoconus AND [artificial intelligence]) OR (keratoconus AND [machine learning]) OR (keratoconus AND algorithms) OR (keratoconus AND [automatic scoring]) OR (keratoconus AND [automatic detection])).

The following sections define the inclusion and exclusion criteria.

Primary research articles, meeting the following requirements were deemed suitable for inclusion in this review:

- Full text original papers that evaluated machine learning algorithms in the diagnosis of KC;
- No limit on the year of publication was applied;
- Publications only in the English language were included;
- Publications in which KC was the only corneal condition evaluated.
- Criteria for exclusion:
- Publications evaluating other corneal diseases without focusing only on KC;
- Publications evaluating the efficacy of machine learning in treating KC (treatments);
- Review papers;
- Publications in which no machine learning algorithms were included, but only statistical research was undertaken;
- Non-English publications.

**Figure 2:**
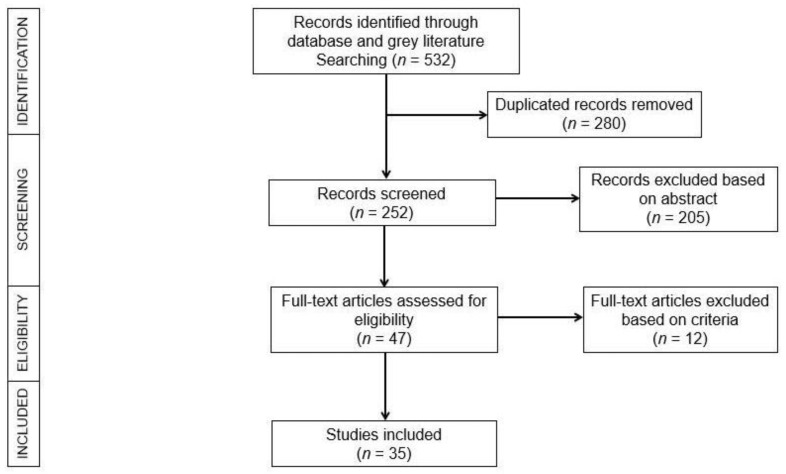
The PRISMA flowchart illustrating the literature selection process.

The review articles were imported into Endnote (version X9), which was used to perform the initial screening. Two independent reviewers (K.C. and S.S.) screened the titles and abstracts of journals for possible qualifying studies, and inconsistencies were settled by consensus. Both reviewers thoroughly analyzed all potential qualifying studies for inclusion/exclusion. (Figure 2).

### 2.2. Data Extraction

A customized analysis form was generated in Microsoft Excel. The following items were reviewed from each study: authors, publication year, country, study objective, sample size, reference standard diagnosis methods, corneal imaging systems used to generate data and machine learning method used, indicators of studies’ quality, and the number of true positives (TP), false positives (FP), true negatives (TN), and false negatives (FN). If the number of TP, FP, TN, or FN were not presented, then these values were derived from the data provided, such as sensitivity and specificity. The meta-analysis omitted studies that lacked data on TP, FP, TN, or FN or that lacked the capacity to measure these data to create a two-way contingency chart.

### 2.3. Reporting Completeness of Machine Learning Studies in KC

We evaluated the reporting completeness of machine learning research in this study by referring to the TRIPOD (transparent reporting of a multivariable prediction model for individual prognosis or diagnosis; www.tripod-statement.org (accessed on 7 June 2021)) statement relevant to model development. This statement contains a 20-item checklist, totaling 31 items when all sub-items are included. The checklist includes questions about the title, abstract, background, methods, results, discussion, supplementary material, and funding information. Two items (5c, i.e., “Give details of treatments received, if relevant”, and 11, i.e., “Provide details on how risk groups were created, if done”) were omitted since they were irrelevant to the research covered in this review. Each study was therefore evaluated on a total of either 28 or 29 possible items. This total number of items varied between 28 and 29 since item 14b (i.e., “If nothing on univariable analysis (in methods or results) is reported, score not applicable”) may be rated as “not applicable” and was thus omitted from the calculation of reporting adherence. If a study had data for several models, we extracted data for the model with the highest performance. Each included item received a score of “1” for adherence and a score of “0” for non-adherence. Multiple items (items 1, 2, 3a, 4b, 5a, 6a, 7a, 7b, 9, 10a, 10b, 10d, 13a, 13b, 14a, 15a, 16, 17, 20 and 22) in the TRIPOD analysis were derived from several sub-items (the sub-items for each number can be found in www.tripod-statement.org (accessed on 7 June 2021)). The score was therefore determined by the combination of several elements rather than a single element. The results of each TRIPOD item for each paper and the level of reporting adherence for each TRIPOD item were documented systematically in a spreadsheet.

For each machine learning study, we calculated the TRIPOD adherence score by dividing the sum of TRIPOD items adhered to by the entire number of applicable TRIPOD items in the study. The average adherence score was calculated using the median value of the adherence score across all studies. For each TRIPOD item, the adherence score was calculated by dividing the number of studies that adhered to the item by the total number of applicable studies for the item. The median value was used to represent the average adherence score for each TRIPOD item.

### 2.4. Statistical Methods

All analyses were configured in ‘mada’ and ‘metafor’ package from RStudio Server Pro (PBC, Boston, MA) (Version 1.3.1056-1) for Windows. To measure the overall machine learning performance for KC detection, the sensitivity and specificity values for all presented models were pooled, following the bivariate meta-analysis method of Reitsma et al. [13] using linear mixed model techniques. The bivariate method retains the two-dimensional nature (sensitivity and specificity) of the original data. Pairs of sensitivity and specificity are jointly evaluated, accommodating any correlation that could exist between sensitivity and specificity using a random effects approach. This was accomplished using the ‘mada’ package (Version 0.5.10) from RStudio. The 95% confidence interval (CI) of the sensitivity and specificity of various imaging systems were compared. The Deeks’ funnel asymmetry test, developed especially for diagnostic test accuracy [14], was used to determine if there was cross-study publishing bias [15].

A hierarchical summary receiver-operating characteristic (HSROC) curve was fitted. Each individual study was presented as a circle and plotted within the HSROC curve. The summary point was represented by a dot surrounded by a 95% confidence interval (95% CI).

### 2.5. Outcomes Measure

The primary outcome indicator was the diagnostic accuracy of machine learning algorithms through a variety of imaging technologies for the identification of KC, as determined by the pooled sensitivity and specificity values. 

## 3. Results

### 3.1. Search Collection

**Figure 3:**
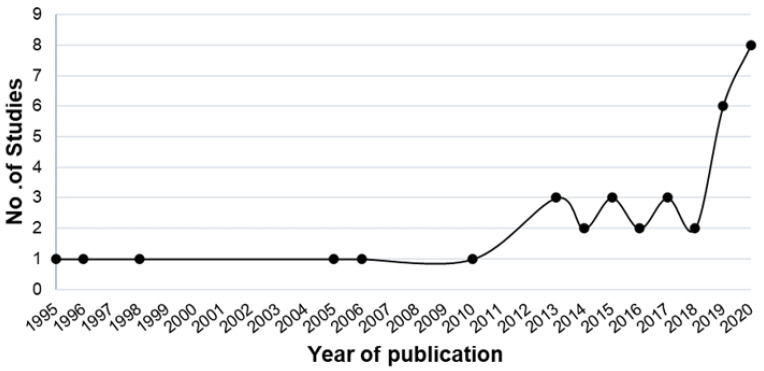
The count of machine learning studies in KC from 1995 to 2020.

Of the initial literature search, 532 studies were retrieved, and 280 duplicates were omitted. Following review of the title, abstract, and full text, 35 studies were included in the review. There was an increasing trend of machine learning studies in KC published over time (Figure 3), with the earliest study published in 1995 by Maeda et al. [12] and increasing to over 30 studies in 2020.

### 3.2. Search Characteristics

The 35 articles on machine learning and KC were reviewed were classified into three categories based on their aims: detecting KC eyes from controls, differentiating early KC from controls, and identifying different KC severities. Each study focused on one or more of these aims, with 12 research papers focused on KC versus control, 4 research papers focused on early KC versus control, 1 article focused on KC severity, and 18 publications with multiple aims. There was no study examining the progression of KC.

Following classification of studies with multiple aims, the following results were obtained: 30 studies [12,16,17,18,19,20,21,22,23,24,25,26,27,28,29,30,31,32,33,34,35,36,37,38,39,40,41,42,43,44] have evaluated machine learning models to distinguish KC from controls, 14 studies [20,21,27,28,29,31,33,37,39,44,45,46,47,48] have evaluated machine learning models to distinguish early KC from controls, and 6 studies [12,33,35,40,42,49] have assessed machine learning models in KC staging. In this review, we utilize the term early KC rather than subclinical KC or forme fruste KC, due to a lack of unified criteria for these terms [20,21,27,28,29,31,33,37,39,44,45,46,47,48,49,50]. Meta-analyses were conducted on each group of studies. A summary of the final 35 studies included in this current study can be found in Table 1.

Of the 35 studies, 33 focused on developing machine learning models, while 2 [16,46] involved external validation of existing models (in a context other than that used for the model development). Four studies [19,22,31,37] developed and externally validated models in the same study (in a separate data set, eliminating random training test splits, and cross-validation), and used data from several centers. The remaining studies analyzed data from a single center (1 study [29] analyzed data from two centers) and internally validated the developed model (e.g., via cross validation, random training test splits). Studies were conducted in different countries, including the United States of America (USA) (*n* = 4) [12,17,18,30], Japan (*n* = 3) [19,42,49], France (*n* = 2) [21,23], the United Kingdom (UK) (*n* = 1) [24], India (*n* = 1) [26], Brazil (*n* = 2) [25,45], Belgium (*n* = 3) [28,33,37], Hungary (*n* = 2) [27,33], Austria (*n* = 1) [41], Australia (*n* = 1) (conducted by our group [48]), Spain (*n* = 3) [35,36,40], Taiwan (*n* = 1) [44], and China (*n* = 2) [39,47], and 4 investigations [22,29,31,34] were cross-ethnicity studies.

### 3.3. Detecting KC from Controls and Meta-Analysis

A total of 26 studies [12,16,17,18,19,20,21,22,23,24,25,26,27,28,29,30,31,32,33,34,35,36,37,38,39,40] developed machine learning models that were based either directly on captured parameters or as calculated parameters from corneal topography or tomography systems. The number of parameters used in these studies ranged from 5 to 443 (Table 1). The machine learning algorithms explored in these studies included decision tree, discriminant analysis, logistic regression, naive bayes, neural networks, random forest, and support vector machine. The majority of these studies (*n* = 23) employed a single machine learning algorithm, while 4 studies [24,29,31,38] compared several algorithms.

Four articles directly analyzed images [41,42,43,44] generated by corneal topography or tomography systems, as opposed to the image parameters. Convolutional neural networks, a common deep learning-based method, were used in all these studies.

Meta-analysis was performed on 26 of the 30 studies, with 4 studies [37,39,41,43] being excluded due to inadequate data needed to quantify the TP, FP, TN, and FN. Asterisks (*) indicated studies that were excluded from the meta-analysis in Table 1. Deeks’ funnel plot (Supplementary Figure S1) was used to assess possible publication bias. No evidence of publication bias was apparent (*p* = 0.91).

The pooled sensitivity and specificity for KC versus control were 0.970 (95% CI 0.949–0.982) and 0.985 (95% CI 0.971–0.993). Pooled performance was computed using a bivariate random-effects model and represents a summary estimate of the sensitivity and specificity values (i.e., TP, FP, TN, and FN) obtained from each individual research [13]. The most frequently employed imaging technologies used were Pentacam (including Pentacam HR), TMS (including TMS-1 and TMS-4), and Orbscan (including Orbscan II and Orbscan IIz). Five studies analyzed data from the Pentacam, four studies from TMS, three studies from Orbscan, and other studies used data from Corvis, GALILEI, Sirius, or Keratron. For the studies based only on Pentacam data, the pooled sensitivity was 0.987 (95% CI 0.971–0.994) and the pooled specificity was 0.989 (95% CI 0.963–0.997). In the case of TMS, the pooled sensitivity was 0.943 (95% CI 0.897–0.969) and the pooled specificity was 0.978 (95% CI 0.954–0.989), whereas for the Orbscan data, the pooled sensitivity was 0.947 (95% CI 0.886–0.976) and the pooled specificity was 0.983 (95% CI 0.917–0.997) (Table 2).

### 3.4. Detecting Early KC from Controls and Meta-Analysis

A total of 13 studies [20,21,27,28,29,31,33,37,39,45,46,47,48] evaluated machine learning models to assess early KC from controls using corneal topography parameters. These studies used fewer parameters compared to those utilized to identify KC in controls, varying from 11 to 55 parameters (Table 1). Ten studies applied a single algorithm and three compared several algorithms (one of which was conducted by our group [48]). Additionally, one study [44] built the model by combining convolutional neural networks and TMS derived images.

Ten studies were included in the meta-analysis, out of a total of fourteen. As shown by an asterisk (*) in Table 1, four studies [37,39,44,45] were omitted from the meta-analysis due to inadequate evidence to calculate the TP, FP, TN, and FN. Assessment of publishing bias with Deeks’ funnel plot (Supplementary Figure S2) found no significant effect (*p* = 0.18).

Overall, the pooled sensitivity was 0.882 (95% CI 0.822–0.923) and pooled specificity was 0.947 (95% CI 0.914–0.967) for early KC versus control. The most widely used imaging machines in the detection of early KC from control eyes were the Pentacam (including Pentacam HR) and Orbscan (including Orbscan II and Orbscan IIz). Six studies used Pentacam data, two studies used Orbscan data, and an additional two studies used data from either GALILEI, or a combination of Pentacam and Corvis. Early KC detection (Table 3) was correlated with a pooled sensitivity of 0.882 (95% CI 0.795–0.935), and a pooled specificity of 0.935 (95% CI 0.874–0.967) for models utilizing Pentacam data. When using Orbscan data, a pooled sensitivity of 0.842 (95% CI 0.504–0.965), and a pooled specificity of 0.958 (95% CI 0.821–0.991) were obtained. 

The pooled diagnostic performance of detecting KC compared to controls was superior to that of early KC with sensitivity (0.970 (95% CI 0.949–0.982) vs. 0.882 (95% CI 0.822–0.923) and specificity (0.985 (95% CI 0.971–0.993) vs. 0.947 (95% CI 0.914–0.967)). This difference implied that early KC detection using machine learning algorithms are still in their infancy.

**Figure 4:**
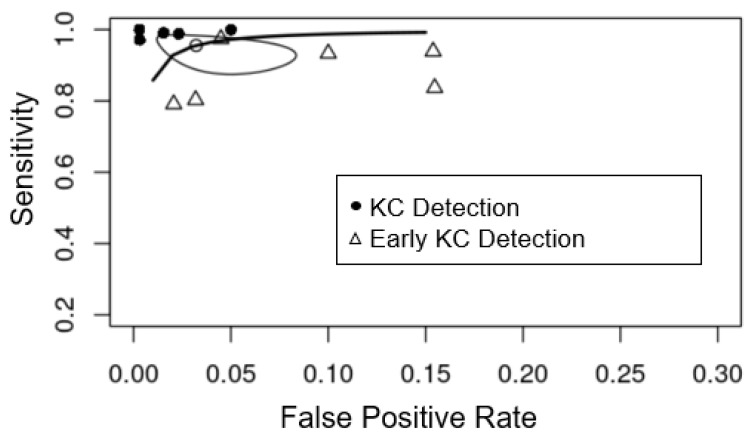
Summary receiver-operating characteristic curves of the diagnostic performance of machine learning detecting KC (black circle) and early KC (triangle) from controls using Pentacam parameters. The white circle is the summary estimate point (sensitivity (0.956 (95% CI 0.897–0.982), specificity (0.968 (95% CI 0.931–0.985)) of studies using Pentacam parameters. The Y-axis represents sensitivity, with higher values indicating greater sensitivity, and the X-axis represents false positive rate, which was equal to 1-specificity, with lower values indicating greater specificity.

The Pentacam tomography system was the most commonly used corneal imaging device for both the KC and early KC categories. In Figure 4, we compare studies that used the Pentacam data set to diagnose KC and early KC. Diagnostic efficiency for detecting KC was superior to that for early KC, as shown by the higher sensitivity (y-axis) and specificity (x-axis) in the plot.

### 3.5. Detection of Different KC Severities

Along with detecting KC eyes as a distinct category, six of the published studies grouped their KC eyes into clinical stages and used machine learning algorithms to identify each stage separately. These studies classified KC eyes into various categories based on a variety of measures. Kamiya et al. [42] classified eyes into Grades 1–4 according to the Amsler–Krumeich (AK) classification scheme, which is mostly focused on keratometry, but often incorporates refraction and pachymetry [51]. Bolarin et al. [35] and Velazquez-Blazquez et al. [40] graded eyes into Grade I–IV plus or mild KC, using a different classification system named RETICS, based on corrected distance visual acuity (CDVA) [51]. Another study, Issarti et al. [33] classified their KC eyes into mild and moderate stages using a self-defined classification (described at the end of Table 4), whereas Maeda et al. [12] did not specify their staging method. Table 4 summarizes these findings. Since no consistent grading system was used for classifying KC severity in these studies—and indeed none is globally established [52]—the findings were therefore not directly comparable. 

The first attempt at proposing a data-driven KC classification scheme was based on density-based clustering by Yousefi et al. [49] using OCT-based parameters from 3156 eyes. They identified five clusters that could represent five stages of KC, ranging from normal to advanced KC.

### 3.6. Reporting Completeness of Machine Learning Studies in KC

**Figure 5:**
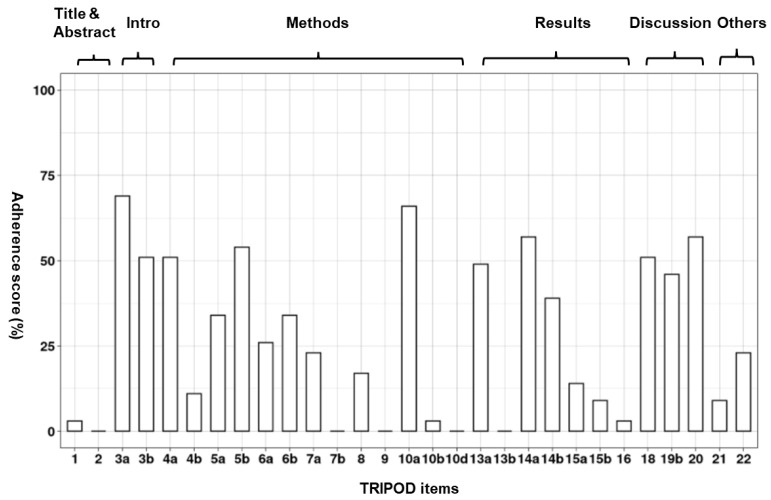
Overall adherence per TRIPOD item.

Publications had only low to moderate adherence to TRIPOD items, ranging between 3% and 48%, with a median of 28%. Each item on the TRIPOD checklist was adhered to by between 0% and 69% of studies, with an average (median) of 23% (Figure 5). Eight items were reported in over 50% of studies, whereas ten items were reported in fewer than 10% of studies. Supplementary Table S1 details results of each TRIPOD item for each paper and the level of reporting adherence for each TRIPOD item. The title, abstract, predictor assessment, management of missing data, model performance assessment, and description of participant characteristics were the most poorly rated items, with no research fulfilling all requirements. In 69% of studies (24/35) [16,17,21,22,23,24,27,30,31,32,33,35,36,37,38,39,40,42,43,44,46,47,48,49], the medical context explanation was well stated, elucidating the reason for creating the models for detecting KC. Moreover, in 66% (23/35) of studies [12,16,17,20,21,22,23,25,28,30,31,32,33,34,36,41,42,43,44,46,47,48,49], the type of transformation of continuous predictors (e.g., linear or nonlinear) were specified.

## 4. Discussion

This is the first comprehensive meta-analysis on KC and machine learning, and it has demonstrated that by using data from a variety of corneal imaging devices, machine learning can reliably distinguish KC eyes from control eyes (pooled sensitivity > 0.90). However, the performance of machine learning models in distinguishing early KC eyes from controls was poorer, with the maximum pooled sensitivity of 0.88. Overall completeness of model was evaluated using the TRIPOD guidelines, and standard reporting compliance was found to be inadequate in all published KC machine learning research so for undertaken.

Machine learning models in KC were developed for a variety of imaging systems. Models themselves are therefore not directly interchangeable owing to the different input expectations. This may have a detrimental effect on the clinical translation of these models. For example, Smadja et al. developed a machine learning model that had a sensitivity of 93.6% and a specificity of 97.2% for discriminating normal eyes from early KC in their study [21]. This model was constructed using the anterior and posterior asphericity asymmetry indices (AAI and OSI), corneal volume, paracentral mean keratometry, and anterior chamber depth derived from a GALILEI machine. Parameters such as AAI and OSI are not accessible in other corneal tomography imaging systems, such as Pentacam [53], suggesting that the generated model cannot be utilized in clinics equipped with the Pentacam system. Pentacam is one of the most frequently used corneal tomographic technologies in clinical practice [11], and our research discovered that the Pentacam is the most frequently utilized source of data in the publications we reviewed [26,27,28,31,33,37,47,48]. As a consequence, despite the fact that the model created by Smadja et al. showed a high level of performance for early KC diagnosis, it is not generally applicable.

The machine learning models that used data from the Pentacam demonstrated a higher pooled sensitivity and specificity in detecting KC and early KC from control eyes compared to other imaging machines. This is likely due to the ability of the Pentacam machine to generate a wider spectrum of data than other systems, including data on the front cornea, the back cornea, corneal pachymetry, and other areas of the anterior eye segment [54,55]. 

A review of the literature on the application of AI to evaluate corneal topography for the diagnosis and early detection of corneal ectasias was recently published [56]. That article summarized significant advances in corneal imaging and the application of AI in KC as viewed by an Eye Care Professional, a biomedical engineer, and a data scientist. It concluded that AI in corneal imaging may improve refractive surgery and diagnosis of corneal ectasias. That review focused exclusively on corneal imaging modalities and their performance in relation to AI. In contrast to that study, our systematic review included meta-analysis-additional information on individual studies as well as the pooled performance of existing machine learning models for detecting KC as well as early KC. In addition, we also reported on the assessment of completeness of items included in the machine learning models using the TRIPOD reporting system. We analyzed each study by its objectives and showed that while substantial research had been undertaken on KC detection, much less had been undertaken on early KC detection, KC severity detection, and detection of progression. Our analysis also evaluated the parameters utilized in prior studies, indicating that no study had analyzed all available parameters despite the extensive use of corneal imaging data. Additionally, we compared and contrasted the corneal imaging systems used. These aspects have not been previously reviewed.

### 4.1. Reporting Completeness of Machine Learning Studies in KC

Only when all model components are completely and transparently reported can the model’s potential clinical usefulness be appropriately assessed. The main objective of machine learning models is to help clinicians in making medical decisions about an individual patient [57]. Users or doctors will need information on the clinical setting in which the diagnosis is needed (e.g., primary care, secondary care, or the general population), as well as the patients for whom the model is appropriate. Additionally, they will need information about which clinical data, referred to as predictors in the model, are necessary for model usage, as well as the definition of the patient outcome to which the model is referring. Unfortunately, this systematic review suggests that the studies on machine learning and KC often lacked sufficient description. Only 34% provided target setting [12,19,24,30,31,33,35,39,42,44,47,49], 23% defined all necessary predictors precisely [17,21,23,25,34,39,45,48], 26% exactly defined the patient outcome [21,28,29,39,44,46,47,48,49], and none completely described participant characteristics (e.g., basic demographics, clinical features, and available predictors).

Attempting to replicate the process of model construction using one’s own data, machine learning researchers working in KC will need to specify information on the approach used to handle any missing data, the full prediction model, and a comprehensive explanation of all model-building procedures. However, only one study (3%) defined the whole model-building process [28], 14% provided the complete prediction model [20,21,26,35,47], and none explained how missing data were handled explicitly.

One factor contributing to the low percentage of adherence is that not all elements are relevant to all machine learning research. For example, it is impractical to mathematically specify the final model in a study using non-regression techniques such as random forest, support vector machine, or neural network. While this is a legitimate rationale, it is important to refer to relevant TRIPOD items and provide as much information about model creation as possible in order to guide the modelling choices and facilitate subsequent validation.

### 4.2. Limitations in the Current Literature

For the studies that reported the number of parameters employed, the range was between 5 and 443. Ruiz et al. used 22 Pentacam parameters to construct a machine learning model for identifying KC and early KC [28]. The parameters they employed, such as corneal curvature and pachymetry, were mostly those found on Pentacam’s four-map selectable report, which is widely used in clinics to assess KC. As noted in their research, these 22 clinically relevant parameters were chosen from a pool of over 1000 accessible from Pentacam. Such pre-modelling parameter selection was observed in the majority of reviewed studies [12,17,18,19,20,21,22,23,24,26,27,29,30,31,32,33,34,35,36,37,38,39,40,42,43,44,45,47,48,49]; although, it was unclear why certain predictors were chosen as inputs. As a consequence of this filtering, the role of unexplored parameters in the identification of KC has remained unclear.

While the majority of research developed novel models for KC detection, only four [19,22,31,37] conducted external validation using other data sources. One common concern is that local data sets used for validation are unlikely to be representative of the target population on a global scale [57]. When evaluated on data sets collected in the United States of America and Switzerland, the machine learning model developed in a cross-ethnic research by Mahmoud et al. [22] performed differently. This implies that most of the current identified models cannot be used in a broad clinical setting since their performance may vary and it will most likely be poorer when applied to other external clinics or nations. We recommend that any model be externally evaluated on a large scale to understand this variation. This is especially true given the relatively small sample sizes reported in each study; international collaboration would therefore be highly advantageous to move this field along.

The development of KC is frequently manifested not only by corneal changes, but also by clinical symptoms such as vision, refraction, and slit-lamp findings. No study has ever incorporated all pertinent data [12,16,17,18,19,20,21,22,23,24,25,26,27,28,29,30,31,32,33,34,35,36,37,38,39,40,41,42,43,44,45,46,47,48,49]. Demographic data, such as age and gender, as well as potential risk factors for KC, such as eye rubbing and family history, may also aid in KC detection. We have limited knowledge of how these critical factors may influence the detection of KC in the machine learning models currently employed. Due to the lack of known risk factors for KC [2], it should be possible to start assessing potential risk factors and include in future machine learning models to assess their importance in diagnosis.

The bulk of reported studies of KC have used data derived from a single corneal topography or tomography imaging device to train their machine learning models. Thus, there is a dearth of information as to what impact combining data from multiple devices would have on machine learning models in the detection of KC. Other forms of data, such as from the corneal epithelial thickness map produced with optical coherence tomography (OCT) and corneal biomechanical measurements, are also increasingly being recognized as crucial in the diagnosis of KC [30], particularly early KC [58]. Integrating data from multiple devices and considering a broader variety of factors may therefore further improve the early detection of KC.

The majority of studies employed a specific machine learning method; although, there was limited information on how or why the authors chose a particular algorithm from a large number of potential alternatives. Given that machine learning algorithms are sometimes referred to as ‘black boxes’, it would be advantageous to evaluate several machine learning algorithms on the same data set and select the best one [59]. This process may assist in avoiding selection bias and may aid in improving early KC detection.

Imbalanced sample sizes were found in about half of the studies analyzed [12,16,20,21,27,28,29,30,31,33,44,46], which could skew the machine learning model and impair its capacity to identify cases. In comparison to the number of control eyes, most studies included a lower number of KC or early KC eyes for model establishment. This may be a concern when considering the performance of the model. For example, in the study by Lopes et al. [31], a random forest model was constructed using 2980 stable (control) eyes and 71 ectatic susceptibility eyes, achieving a sensitivity of 80% and a specificity of 96.8%. However, their model’s baseline accuracy, defined as the ability to identify all eyes in the control group without constructing a model, was 97.7%. Although the analysis obtained a high specificity of 96.8%, this could be deemed as suboptimal in comparison to its potential baseline accuracy. Instead of utilizing accuracy, sensitivity, and specificity, precision value (also known as positive predictive value) may provide a more interpretable evaluation for models with unbalanced sample sizes. This metric has been employed in several studies to evaluate their models [22,28,32,33,36,47,48].

There have been fewer studies on identifying the different stages of KC, and a standardized classification system for KC has yet to be devised. There has been no machine learning study using longitudinal data on KC progression. While applications of machine learning have benefited in predicting and detecting progression indicators in AMD, diabetic retinopathy, and glaucoma [60,61,62,63,64]. The combination of machine learning methods and large clinical data sets may assist in the analysis of KC progression.

### 4.3. Approach for Future Studies

Machine learning has been increasingly used in KC over the last three decades, mostly for the identification of KC and early KC. The advantage of machine learning is that it allows consistent and unbiased diagnosis, which is critical when diagnosing patients at an early stage, as early intervention using treatments such as corneal crosslinking (CXL), could delay or slow disease progression, thus preventing the need for a possible corneal transplant.

There is still room to improve the efficiency of machine learning models in detecting early KC. This may be accomplished by allowing the use of all publicly available data, including complete databases from corneal imaging systems, clinical data, genetic data, and other risk factors. There is also a need to maximize the potential of machine learning techniques by optimizing their output at the methodology and data space levels.

Currently, there are no successful examples of machine learning models being used in clinical practice. This may be due to a lack of large patient populations to validate results, the utilization of various imaging devices, a local participant group comprised of individuals of various ethnic backgrounds, clinicians’ overall acceptance of machine learning techniques for diagnosis and their relative reliability to humans. External model validation on a diverse patient population, as well as the creation of platform-independent models that can be generalized through several corneal imaging systems, are therefore needed. Finally, research on machine learning in KC should also address additional research gaps in the area, such as classifying KC severity, and identifying and forecasting KC progression.

## 5. Conclusions

We present an up to date, comprehensive review on the use of machine learning in KC detection and identify the substantial limitations that need to be overcome to make the diagnostic process more efficient for early keratoconus. In light of our findings on pooling detection performance and low adherence to the TRIPOD checklist, we believe that both improved machine learning model performance in early KC detection and improved quality machine learning research in KC is sorely needed. Despite various challenges, the future of integrating machine learning technologies into clinical practice is promising with the advent of advanced imaging modalities. Machine learning can be further investigated for broad application to the entire process of KC detection and management. In particular, some open avenues for research include early KC detection, risk factor evaluation, prediction of progression, and clinical management guidance. However, global collaboration is essential to obtain larger data sets and more robust models.

## Figures and Tables

**Table 1:** Identified studies using machine learning in detection of KC and early KC.

Study Objectives	First Author	Year	No. of Centers Involved (Country)	Sample Size	No. of KC/Early KC Eyes	Machine Learning Method/s Used	Data Type (No. of Parameters)	Corneal Imaging Modality	Evaluation Methods
Detect KC eyes from controls	Maeda et al. [12]	1995	1 (USA)	176	44	Combined discriminant analysis and classification tree	P (8)	TMS-1	Internal
	Kalin et al. [16]	1996	NR	106	5	Combined discriminant analysis and classification tree	P (8)	TMS-1	Validation study
	Rabinowitz et al. [17]	1998	1 (USA)	241	99	Linear discriminant analysis	P (5)	TMS-1	Internal
	Twa et al. [18]	2005	NR (USA)	244	112	Decision tree	P (36)	Keratron	Internal
	Bessho et al. [19]	2006	2 (Japan)	165	63	logistic regression	P (na)	Orbscan II	External
	Saad et al. [20]	2010	NR	143	31	Discriminant analysis	P (51)	Orbscan IIz	Internal
	Smadja et al. [21]	2013	1 (France)	325	148	Decision tree	P (55)	GALILEI	Internal
	Mahmoud et al. [22]	2013	3 (Colombia, USA, Switzerland)	407	163	logistic regression	P (na)	GALILEI	External
	Saad et al. [23]	2014	1 (France)	166	64	Discriminant analysis	P (7)	Orbscan IIz	Internal
	Silverman et al. [24]	2014	1 (UK)	204	74	Multiple methods	P (161)	Artemis-1	Internal
	Koprowski et al. [25]	2015	1 (Brazil)	746	477	Decision tree	P (11)	Corvis	Internal
	Shetty et al. [26]	2015	1 (India)	128	85	Logistic regression	P (na)	Pentacam	Internal
	Kovacs et al. [27]	2016	1 (Hungary)	120	60	Neural network	P (na)	Pentacam HR	Internal
	Ruiz et al. [28]	2016	1 (Belgium)	648	454	Support vector machine	P (22)	Pentacam HR	Internal
	Ambrosio et al. [29]	2017	2 (Brazil, Italy)	756	276	Multiple methods	P (na)	Pentacam HR & Corvis ST	Internal
	Silverman et al. [30]	2017	1 (USA)	141	30	Discriminant analysis	P (240)	Artemis-1 & Pentacam	Internal
	Lopes et al. [31]	2018	5 (UK, Brazil, Italy, USA)	3648	370	Multiple methods	P (na)	Pentacam	Internal & External
	Chandapura et al. [32]	2019	NR	439	218 including 102 early KC	Random forest	P (27)	Pentacam & OCT	Internal
	* Dos Santos et al. [41]	2019	1 (Austria)	142	70	Convolutional neural network	I	OCT	Internal
	Issarti et al. [33]	2019	1 (Belgium)	624	312	Neural network	P (28)	Pentacam	Internal
	Kamiya et al. [42]	2019	1 (Japan)	543	304	Convolutional neural network	I	AS-OCT	Internal
	* Lavric et al. [43]	2019	NR	3000	1500	Convolutional neural network	I	SyntEyes model	Internal
	Leão et al. [34]	2019	2 (Brazil, Italy)	574	223	Discriminant analysis	P (na)	Corvis ST	NR
	Bolarin et al. [35]	2020	1 (Spain)	169	107	logistic regression	P	Sirius	Internal
	Castro-Luna et al. [36]	2020	1 (Spain)	60	30	Naive Bayes	P	CSO	Internal
	* Issarti et al. [37]	2020	2 (Belgium)	812	508	Neural Network	P (90)	Pentacam HR	Internal & External
	Kuo et al. [44]	2020	1 (Taiwan)	326	170	Convolutional neural network	I	TMS-4	Internal
	Lavric et al. [38]	2020	NR	3151	1181 including 791 early KC	Multiple methods	P (443)	SS-1000 CASIA OCT	Internal
	* Shi et al. [39]	2020	1 (China)	121	38	Neural network	P (49)	UHR-OCT & Pentacam HR	Internal
	Velazquez-Blazquez et al. [40]	2020	1 (Spain)	178	104 including 61 early KC	Logistic regression	P (27)	Sirius	Internal
Detect early KC eyes from controls	Saad et al. [20]	2010	NR	143	40	Discriminant analysis	P (51)	Orbscan IIz	Internal
	Smadja et al. [21]	2013	1 (France)	224	47	Decision tree	P (55)	GALILEI	Internal
	* Ventura et al. [45]	2013	NR (Brazil)	204	68	Neural network	P (41)	Ocular Response Analyzer	Internal
	Chan et al. [46]	2015	1 (Singapore)	128	24	Discriminant analysis	P (na)	Orbscan IIz	Validation study
	Kovacs et al. [27]	2016	1 (Hungary)	75	15	Neural network	P (na)	Pentacam HR	Internal
	Ruiz et al. [28]	2016	1 (Belgium)	261	67	Support vector machine	P (22)	Pentacam HR	Internal
	Ambrosio et al. [29]	2017	2 (Brazil, Italy)	574	94	Multiple methods	P (na)	Pentacam HR & Corvis ST	Internal
	Xu et al. [47]	2017	1 (China)	363	77	Discriminant analysis	P (na)	Pentacam HR	Internal
	Lopes et al. [31]	2018	5 (UK, Brazil, Italy, USA)	3537	259	Multiple methods	P (na)	Pentacam	Internal & External
	Issarti et al. [33]	2019	1 (Belgium)	389	77	Neural network	P (28)	Pentacam	Internal
	Cao et al. [48]	2020	1 (Australia)	88	49	Multiple methods	P (11)	Pentacam	Internal
	* Issarti et al. [37]	2020	2 (Belgium)	812	117	Neural Network	P (90)	Pentacam HR	Internal & External
	* Kuo et al. [44]	2020	1 (Taiwan)	354	28	Convolutional neural network	I	TMS-4	Internal
	* Shi et al. [39]	2020	1 (China)	121	33	Neural network	P (49)	UHR-OCT & Pentacam HR	Internal
KC Severity	Yousefi et al. [49]	2018	multi-center (Japan)	3156		Density-based clustering	P (420)	CASIA OCT	NA

Study objectives: The aim of the research. It was either detecting KC from controls or detecting early KC from controls in this study. No. of Centers involved: The number of centers involved is reported, NR indicated the center is not reported explicitly. Data type (No. of parameters): The kind of data used as inputs to machine learning models. It was either images (graphics) or parameters in this study (numeric). The letter ‘P’ denoted parameters, while the letter ‘I’ denoted images. Corneal Imaging modality: Where the imaging system/systems of the input data was derived. Evaluation methods: Described how the model’s performance was determined. External (evaluation in an independent database), internal (bootstrap validation, cross validation, random training test splits, temporal splits). Asterisks (*) indicated studies that were excluded from the meta-analysis.

**Table 2:** Diagnostic performance of artificial intelligence in detection of KC versus controls using different imaging modalities.

Imaging Modalities	Pooled Sensitivity	Pooled Specificity
Pentacam (*n* = 5)	0.987 (95% CI 0.971–0.994)	0.989 (95% CI 0.963–0.997)
TMS (*n* = 4)	0.943 (95% CI 0.897–0.969)	0.978 (95% CI 0.954–0.989)
Orbscan (*n* = 3)	0.947 (95% CI 0.886–0.976)	0.983 (95% CI 0.917–0.997)
Pooled total (*n* = 26)	0.970 (95% CI 0.949–0.982)	0.985 (95% CI 0.971–0.993)

**Table 3:** Diagnostic performance of machine learning on detection early KC using different imaging modalities.

Imaging Modalities	Pooled Sensitivity	Pooled Specificity
Pentacam (*n* = 6)	0.882 (95% CI 0.795–0.935)	0.935 (95% CI 0.874–0.967)
Orbscan (*n* = 2)	0.842 (95% CI 0.504–0.965)	0.958 (95% CI 0.821–0.991)
Pooled total (*n* = 10)	0.882 (95% CI 0.822–0.923)	0.947 (95% CI 0.914–0.967)

**Table 4:** Characteristics of machine learning-assisted studies for detection of KC severity.

First Author	Year	Severity Grading (No. of Eyes)	Definition/Classification Methods	Corneal Imaging Modality	Reported Sensitivity in Detection of Each Severity Level
Maeda et al. [12]	1995	Mild (15) Moderate (18) Advanced (11)	NA	TMS-1	Mild: 100% Moderate: 100% Advanced: 91%
Kamiya et al. [42]	2019	Grade 1 (108) Grad e2 (75) Grade 3 (42) Grade 4 (79)	Amsler–Krumeich classification	AS-OCT	Grade 1: 88.9% Grade 2: 68% Grade 3: 71.4% Grad e4: 74.7%
Issarti et al. [33]	2019	Mild KC (220)	^a^ Self-defined	Pentacam	98.81%
Issarti et al. [33]	2019	Moderate KC (229)	^b^ Self-defined	Pentacam	99.91%
Bolarin et al. [35]	2020	Grade I (44) Grade II (18) Grade III (15) Grade IV (15) Grade IV plus (15)	RETICS grading	Sirius	Grade I: 59.1% Grade II: 33.3% Grade III: 40% Grade IV: 80% Grade IV plus: 86.7%
Velazquez-Blazquez et al. [40]	2020	Mild KC (42)	RETICS grading	Sirius	Mild KC: 63%

^a^ A clear cornea, tomography maps compatible with KC, a Fleischer ring at the apex base, slight thinning, and anterior and/or posterior corneal steepening; ^b^ Slit-lamp findings compatible with KC, corneal thinning at the apex, Vogt striae, a clearly visible Fleischer ring and corneal tomography compatible with KC; The severity of KC was considered to be increasing from Grade 1 to Grade 4 and for Grade I to Grade IV plus.

## Data Availability

The corresponding author had full access to all the data in the study; data are available upon reasonable request.

## Supplementary Materials

The following supporting information can be downloaded at: https://www.mdpi.com/article/10.3390/jcm11030478/s1, Figure S1: Assessment of publication risk bias across studies using Deeks’ funnel plot asymmetry test in detecting KC from controls. ESS, effective sample size, which is determined as a function of the number of diseased (n1) and non-diseased (n2) subjects: (4n1 * n2)/(n1 + n2). The y-axis shows the inverse of the square root of the effective sample size (1/root(ESS)). The x-axis shows the natural logarithm of the diagnostic odds ratio (lnDOR (TP * TN)/(FP * FN)). In this figure, the vertical line represents the meta-analysis summary estimate, and each black circle represents one study, and dispersed symmetrically right and left the vertical line implies a low probability of publication bias for the included research. The dotted line indicates the ESS weighted regression tests of funnel plot asymmetry, and *p* values < 0.05 were considered as significant; Figure S2: Assessment of publication risk bias across studies using Deeks’ funnel plot asymmetry test in detecting early KC from controls. ESS, effective sample size, which is determined as a function of the number of diseased (n1) and non-diseased (n2) subjects: (4n1 * n2)/(n1 + n2). The y-axis shows the inverse of the square root of the effective sample size (1/root(ESS)). The x-axis shows the natural logarithm of the diagnostic odds ratio (lnDOR (TP * TN)/(FP * FN)). In this figure, the vertical line represents the meta-analysis summary estimate, and each black circle represents one study, and dispersed symmetrically right and left the vertical line implies a low probability of publication bias for the included research. The dotted line indicates the ESS weighted regression tests of funnel plot asymmetry, and *p* values < 0.05 were considered as significant; Table S1: Results of each TRIPOD item for each paper and the level of reporting adherence for each TRIPOD item.
