# Supplementary figures and images for: Accuracy of Machine Learning Assisted Detection of Keratoconus: A Systematic Review and Meta-Analysis
Source: J Clin Med. 2022 Jan 18;11(3):478. doi: 10.3390/jcm11030478 (PMC8836961; doi:10.3390/jcm11030478)

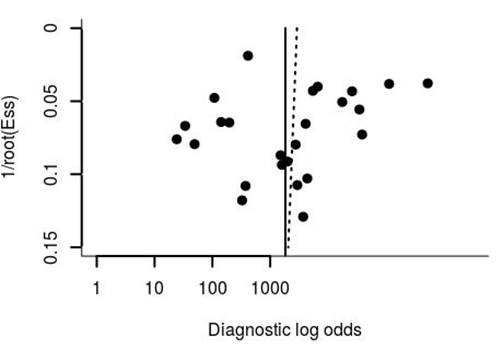

Supplement: Supplementary file 1 [file jcm-11-00478-s001.zip › Supplementary/Supplementary Figure 1.tif]

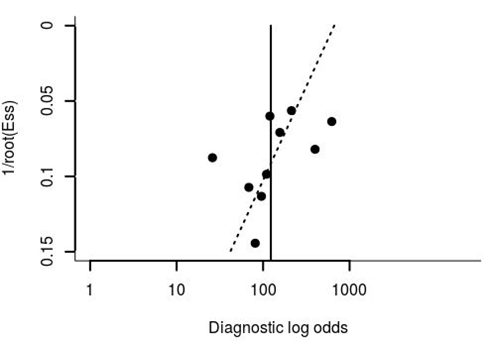

Supplement: Supplementary file 1 [file jcm-11-00478-s001.zip › Supplementary/Supplementary Figure 2.tif]
